# Current Status and Prospects of Anesthesia and Breast Cancer: Does Anesthetic Technique Affect Recurrence and Survival Rates in Breast Cancer Surgery?

**DOI:** 10.3389/fonc.2022.795864

**Published:** 2022-02-09

**Authors:** Ryungsa Kim, Ami Kawai, Megumi Wakisaka, Takanori Kin

**Affiliations:** ^1^ Department of Breast Surgery, Hiroshima Mark Clinic, Hiroshima, Japan; ^2^ Department of Breast Surgery, Hiroshima City Hospital, Hiroshima, Japan

**Keywords:** breast cancer, anesthetic technique, recurrence, survival, immune response

## Abstract

The relationship between the anesthetic technique and cancer recurrence has not yet been clarified in cancer surgery. Surgical stress and inhalation anesthesia suppress cell-mediated immunity (CMI), whereas intravenous (IV) anesthesia with propofol and regional anesthesia (RA) are known to be protective for CMI. Surgical stress, general anesthesia (GA) with inhalation anesthesia and opioids contribute to perioperative immunosuppression and may increase cancer recurrence and decrease survival. Surgical stress and GA activate the hypothalamic-pituitary-adrenal axis and release neuroendocrine mediators such as cortisol, catecholamines, and prostaglandin E_2_, which may reduce host defense immunity and promote distant metastasis. On the other hand, IV anesthesia with propofol and RA with paravertebral block or epidural anesthesia can weaken surgical stress and GA-induced immunosuppression and protect the host defense immunity. IV anesthesia with propofol and RA or in combination with GA may reduce cancer recurrence and improve patient survival compared to GA alone. We review the current status of the relationship between anesthesia and breast cancer recurrence using retrospective and prospective studies conducted with animal models and clinical samples, and discuss the future prospects for reducing breast cancer recurrence and improving survival rates in breast cancer surgery.

## Introduction

Over the past two decades, the relationship between anesthesia and cancer recurrence has been a controversial issue in the field of oncological surgery because surgical stress and intraoperative anesthesia impair host immunity (1). The first report on anesthesia and cancer recurrence, published in 2000, describes a retrospective analysis of patients with cutaneous melanoma (2). In that study, the survival rate of patients who received local anesthesia (LA) was higher than that of patients who received general anesthesia (GA), suggesting that LA reduces the recurrence of melanoma relative to GA (2). This finding reflects the impairment of cell-mediated immunity (CMI) and host immune responses by inhalation GA (3). Indeed, several preclinical models have shown that inhaled anesthetics inhibit natural killer (NK) cell– and T lymphocyte-mediated immunity, resulting in increased metastasis (4, 5). Immunosuppression by inhalation anesthesia is mediated by the stimulation of the hypothalamic–pituitary–adrenal (HPA) axis, which releases neuroendocrine mediators such as catecholamines, prostaglandin E_2_ (PGE_2_), cytokines, and cortisol. Other neuroendocrine mediators, such as interleukin 6 (IL-6) and matrix metalloproteinases (MMPs), are also secreted and play critical roles in the regulation of tumor growth and angiogenesis (6). The impairment of CMI may reactivate micrometastases that are already disseminated at the time of surgery, increasing the frequencies of cancer recurrence and distant metastasis (6). In contrast, LA allows the maintenance of spontaneous breathing during surgery and has a weaker immunosuppressive effect than does GA (7).

Other factors that can cause immunosuppression during cancer surgery include surgical stress and opioid use. Surgical stress is limited by the size of the operative field, duration of the operation, and amount of blood loss (8). Opioids are commonly used in combination with inhalation anesthetics as analgesics and sedatives for GA, but non-synthetic and synthetic opioids can suppress CMI, depending on the dose and duration of use (9). In contrast, intravenous anesthesia (IVA) with propofol protects CMI (4, 10), as does regional anesthesia (RA) with paravertebral block (PVB) or epidural anesthesia. RA blocks afferent neurotransmitter pathways from peripheral nerves to the central nervous system and the efferent activation of the sympathetic nervous system (SNS), thereby reducing the release of neuroendocrine mediators such as glucocorticoids and allowing the minimization of opioid use (11).

Retrospective studies of anesthesia and cancer recurrence have yielded positive and negative results, depending on the type of cancer and the anesthetic technique used. Several prospective randomized controlled trials (RCTs) are underway, and preliminary results suggest that the effects of anesthesia on cancer recurrence and survival differ depending on the type of cancer. In this review, we examine the effect of the anesthetic technique used during breast cancer surgery on breast cancer recurrence and survival, and discuss the current status of and future prospects for anesthesia and breast cancer.

## Effects of Surgical Stress and Anesthesia on Immune Function and Breast Cancer Progression

**Figure 1:**
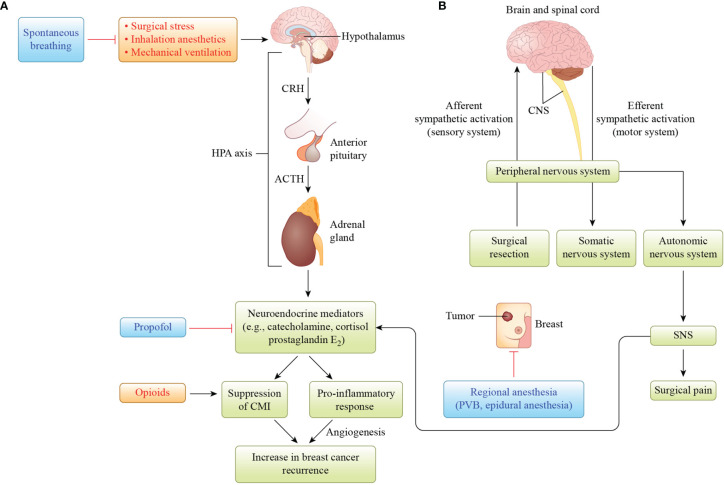
Illustration of the hypothesis that hypothalamus–pituitary–adrenal (HPA) axis and sympathetic nervous system (SNS) activation by surgical stress, inhalation anesthetics, and mechanical ventilation is involved in increased breast cancer recurrence. **(A)** Activation of the HPA axis results in the release of neuroendocrine mediators such as catecholamine, cortisol, and prostaglandin E_2_. These mediators suppress cell-mediated immunity (CMI), resulting in host immunosuppression, and produce pro-inflammatory cytokines to induce angiogenesis, which has been associated with increased breast cancer recurrence. Propofol protects against CMI suppression mediated by neuroendocrine mediators, whereas opioids suppress CMI. **(B)** When breast cancer surgery activates the afferent nervous system from the peripheral to the central nervous system (CNS), it activates the efferent nervous system from the CNS to the peripheral nervous system, autonomic nervous system, and sympathetic nervous system (SNS), which releases neuroendocrine mediators. Regional anesthesia, such as paravertebral blockade (PVB), or epidural anesthesia inhibits the SNS-induced release of neuroendocrine mediators. Reciprocal activation of the HPA axis and SNS by surgical stress and/or inhalation anesthesia may increase breast cancer recurrence. CRH, corticotropin-releasing hormone; ACTH, adrenocorticotropic hormone.

**Figure 2:**
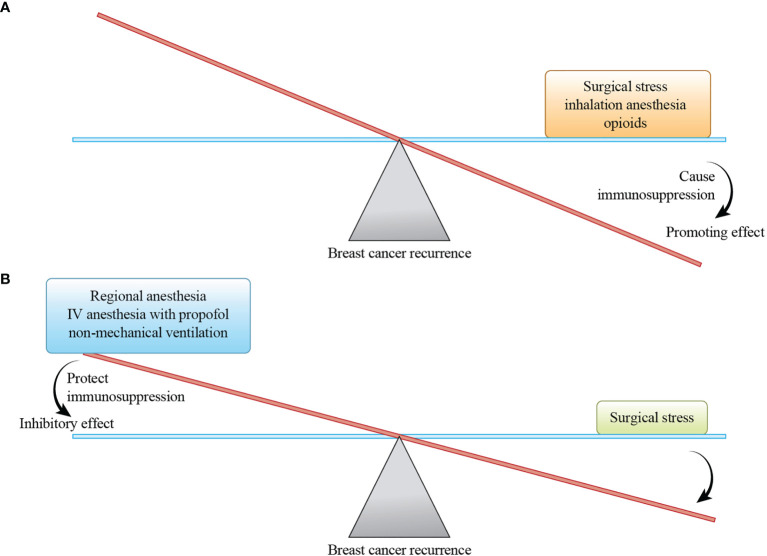
A hypothetical balance of recurrence-promoting and -inhibiting factors related to breast cancer surgery. The magnitude of the promoting effect depends on the size of the breast cancer surgery, and the magnitude of the inhibitory effect depends on the inhibiting factors selected. **(A)** Surgical stress, inhalation anesthesia, and opioids promote breast cancer recurrence by causing immunosuppression. **(B)** Regional anesthesia, intravenous (IV) anesthesia with propofol, and non-mechanical ventilation reduce breast cancer recurrence by protecting immunosuppression.

Stress caused by surgery and anesthetics is believed to trigger changes in the immune system, the host defense, and tumor formation. The constellation of anesthesia, stress, and immunosuppression effects on breast cancer recurrence is illustrated in **Figure 1**. The hypothetical balancing of recurrence-promoting and -inhibiting factors related to breast cancer surgery is shown in **Figure 2**.

### Surgical Stress

In general, the invasiveness of surgery, postoperative pain, and intraoperative bleeding are stress factors in cancer surgery. For thoracic and abdominal surgeries, long operative times, excessive invasiveness, and massive blood loss are major stress factors leading to decreased immunity in patients with cancer. As surgery alters the microenvironments of the nervous, endocrine, inflammatory, and immune systems (12), the stress response induced by surgery may activate angiogenesis and promote tumor growth (13–15). Breast cancer surgery types are breast-conserving surgery (BCS), mastectomy (MT) with or without subsequent reconstruction, sentinel lymph-node biopsy (SLNB), and axillary lymph-node dissection (ALND). BCS is less invasive than MT and yields higher survival rates (16–18), and SLNB is less invasive than ALND. These surgeries usually take 1–2 hours, and those that do not involve reconstruction cause less blood loss. Relative to thoracic and abdominal surgeries, breast cancer surgery is minimally invasive due to its de-escalation based on the concept that breast cancer is a systemic disease, and to the development of adjuvant and neoadjuvant chemotherapies. Nevertheless, surgical resection, even in patients with breast cancer, can increase the expression of MMP-9 and vascular endothelial growth factor (VEGF), which may promote tumor growth and metastasis, as documented in some xenograft models of breast cancer (19). Plasma VEGF levels are increased by surgical stress during MT (13), and plasma transforming growth factor-β levels have been shown to increase and to be associated with lung metastasis after MT in animal models (20). In patients with breast cancer, the acceleration of metastasis due to the proliferation of distant and dormant micrometastases after surgical resection has been observed (21).

### Inhalation Anesthesia

In anesthesia-induced immunosuppression, inhalation anesthetics such as sevoflurane suppress CMI and promote tumor cell proliferation and angiogenesis. Sevoflurane induces the apoptosis of T lymphocytes and upregulates the expression of hypoxia-inducible factor-1α (HIF-1α) *in vitro*; other inhalation anesthetics, including isoflurane and desflurane, upregulate HIF-1α expression *in vitro* and *in vivo* (5, 22). Sevoflurane has also been shown to increase the levels of MMP-3 and -9 in patients undergoing breast cancer surgery (23). Surgical stress and inhalation anesthesia may increase distant metastasis in patients with cancer by activating the HPA axis and the SNS *via* the release of neuroendocrine mediators such as cortisol, catecholamines, and PGE_2_. Sevoflurane increases the proliferation, migration, and invasion of estrogen receptor (ER)-positive and -negative breast cancer cells (24). Furthermore, serum from patients who received propofol and PVB, but not from those who received sevoflurane and opioids, for breast cancer surgery inhibited the growth of ER-negative breast cancer cells *in vitro* (25). On the other hand, a recent study showed that sevoflurane, especially at high concentrations, inhibits the migration, invasion, and epithelial–mesenchymal transition (EMT) of breast cancer cells, mediated by the upregulation of micro-RNA (miR)-139-5p and down-regulation of adenosine diphosphate–ribosylation factor 6 (ARF6) due to miR-139-5p–ARF6 binding *in vitro* (26). These effects are based on the involvement of miR-139-5p in the metastatic processes of breast cancer cell migration and invasion, and the key functional role of ARF6 in tumor angiogenesis (27, 28).

### Opioids

Opioids such as morphine stimulate the growth of tumor cells *in vitro*, and synthetic opioids such as fentanyl and remifentanil also inhibit CMI. Most opioids inhibit the proliferation of T lymphocytes (29). Morphine inhibits NK cell cytotoxicity and T cell proliferation and differentiation, promotes T lymphocyte apoptosis, and decreases the expression of the lipopolysaccharide receptor toll-like receptor 4 on macrophages *in vitro* and *in vivo* (29–32). Similarly, fentanyl was found to decrease NK cell cytotoxicity, resulting in lung metastasis, in an animal model (33), but to increase regulatory T cell (Treg) expression in patients who had undergone breast cancer surgery (34). Remifentanil has also been shown to inhibit NK cell cytotoxicity and T lymphocyte proliferation in a rat model (35). Opioid analgesics may affect tumor development by modulating cell proliferation and cell death (36–38). Various immunocompetent cells express μ-opioid receptors (MORs) and induce apoptosis under opioid alkaloid treatment, suggesting that opioids suppress the immune response (39). In contrast, the overexpression of MORs, which promotes tumor growth and metastasis, has been observed in several human cancers (40).

The tumor growth–promoting effects of opioids are mediated by a signaling cascade involving Akt and extracellular signal–regulated kinase (ERK), whereas their death-promoting effects are mediated by the inhibition of nuclear factor-κB (NF-κB), increased expression of Fas, stabilization of p53, and activation of p38 and c-Jun-N-terminal kinase (39). In a recent study, morphine promoted angiogenesis and tumor cell proliferation in recurrent breast tumors in nude mice after breast cancer surgery, likely with the involvement of the PI3K/c-Myc signaling pathway (41). In a triple-negative (TN) breast cancer xenograft model, morphine promoted TN breast cancer metastasis and angiogenesis, and the non-steroidal anti-inflammatory drug (NSAID) ketorolac inhibited these effects, possibly due to its enhancement of thrombospondin-1 synthesis and inactivation of the PI3K/Akt/c-Myc pathway (42).

Opioid-induced cell proliferation and cell death are thought to depend on the opioid concentration and duration of exposure. *In vitro*, low concentrations and single doses of opioids promote tumor growth, whereas chronic use and high opioid concentrations inhibit this growth (43). Clinically useful doses of morphine have been shown to promote tumor neovascularization and progression in xenograft models of human breast cancer (38), and to promote angiogenesis and the progression of ER-negative breast cancer *in vitro* and *in vivo* (44). Morphine also stimulates the proliferation of vascular endothelial cells, which is mediated by the mitogen-activated protein kinase pathway, *in vitro* (45). MORs are thought to play important roles in angiogenesis and carcinogenic signaling. On the other hand, the preoperative and postoperative use of morphine as analgesia was found to decrease the tumor-promoting effects of surgery (46) and to significantly suppress the surgery-induced increase in corticosterone production (47) in rat models. These results suggest that preoperative morphine administration plays an important role in the prevention of surgery-induced metastasis. Indeed, a recent study showed that increases in intraoperative opioid doses improved recurrence-free survival (RFS), but not overall survival (OS), in patients with TN breast cancer (48). The authors explained this effect by noting that the expression of opioid receptors in tumor and immune cells was consistent with the protective effect of opioid agonists, with no or decreased expression of protumor receptors and elevated expression of antitumor receptors (48).

### Tramadol and Dexmedetomidine

Analgesic use after breast cancer surgery may also affect long-term outcomes. Tramadol is an atypical opioid analgesic that has shown antitumor effects on breast cancer cells *in vitro* and *in vivo* (49, 50). The mechanism by which tramadol exerts these effects involves cell cycle arrest and the induction of apoptosis *via* ERK, due to the decreased expression of 5-hydroxytryptamine_2B_ receptor and transient receptor potential vanilloid-1, as demonstrated by *in-vitro* experiments (49). *In vivo*, tramadol administration decreased the expression of inflammatory cytokines such as IL-6 and tumor necrosis factor-α (TNF- α), which are involved in tumor growth and invasion, and maintained NK cell activity, unlike morphine (50). Tramadol activates the host immune system by increasing lymphocyte proliferation and NK cell activity in patients with cancer (29). Furthermore, a retrospective analysis showed that tramadol use was associated with reduced breast cancer recurrence and mortality in patients who had undergone breast cancer surgery (49).

Dexmedetomidine (DEX) is a selective α2 adrenergic receptor agonist that has analgesic and antiemetic effects and can be used as an anesthetic adjuvant in cancer surgery. An RCT conducted to evaluate the effect of DEX on perioperative immune function in patients undergoing MT showed that DEX maintains the host immune function, as reflected in the expression of immune cells such as CD4/8 and NK cells, and cytokines such as IL-2, IL-6, and IL-10 (51). Furthermore, a meta-analysis showed that the use of DEX as an adjuvant to anesthetics reduces the use of analgesics such as tramadol, morphine, and fentanyl; prolongs the time to patients’ first analgesic request; and relieves postoperative pain (52). The mechanism by which DEX exerts its analgesic effect is unclear, but it may be related to the decreased expression of inflammatory cytokines such as IL-6, TNF-α, and C-reactive protein (53). Furthermore, DEX administration has been shown to enhance host protective immunity, including increases in NK and CD4+ cells and CD4/CD8 and T helper cell (Th)1/Th2 ratios, *via* suppression of the HPA axis and SNS stimulation of the surgical stress response in the setting of cancer surgery (53). Despite its anti-inflammatory effects, however, DEX has also been reported to be tumor promoting. It was shown to promote breast cancer cell proliferation, migration, and invasion *via* activation of the α2B adrenergic receptor/ERK signaling pathway *in vitro* and *in vivo* (54), and to promote the metastasis of breast, lung, and colon cancer cells, mediated by the α2 adrenergic receptor, in animal models (55).

### Regional and Intravenous Anesthesia

RA (e.g., PVB and epidural anesthesia) is expected to suppress neuroendocrine stress responses, reduce the need for opioids, decrease immunosuppression, and induce antitumor and anti-inflammatory responses, contributing to the reduction of cancer recurrence, due to the effects of LA on the whole body. Clinical trials suggest that the use of RA and avoidance of opioids is beneficial, but the isolated benefits of abstaining from opioids and adding RA are unclear.

IVA with propofol does not suppress CMI, but it increases cytotoxic T lymphocyte (CTL) activity, decreases inflammatory cytokine levels, and suppresses cyclooxygenase 2 and PGE_2_ functions (10, 56, 57). The *in-vitro* activity of CTLs against EL4 tumor cells was significantly greater after propofol injection than after the injection of vehicle (Intralipid; Nihon Pharmaceutical, Co., Ltd., Osaka, Japan) or saline (10). Propofol also inhibited the growth of EL4 tumors inoculated into mice, suggesting that it has an immune-mediated antitumor effect (10). Propofol and lidocaine reduced lung metastasis, whereas methylprednisolone increased such metastasis, in a mouse model of breast cancer surgery under sevoflurane anesthesia (56).

Propofol maintains the host immune defense *via* NK cells and innate immunity, and may increase the survival rate of patients with breast cancer more effectively than do inhalational anesthetics (58). Propofol is thought to have antitumor and tumor-promoting effects, depending on its concentration (58). It has been found to inhibit breast tumor invasion and migration by affecting the expression of MMPs, enzymes that play important roles in the degradation of extracellular proteins and EMT (59), *via* NF-κB inhibition *in vitro* (60). In another *in-vitro* study, propofol inhibited the migration, but not proliferation, of ER-positive and -negative breast cancer cells, mediated by decreased expression of neuroepithelial transforming gene 1, which is associated with enhanced migration (61). *In-vitro* studies have shown that propofol induces apoptosis in breast cancer cells, by decreasing miR-24 expression and increasing the expression of p27 and cleaved caspase-3 (62), and by increasing the expression of pro-apoptotic proteins such as Bax, Bak, and cytochrome c, followed by the activation of the caspase cascade through an intrinsic apoptotic signaling pathway mediated by reactive oxygen species (63). In addition, propofol was shown in an *in-vitro* study to suppress HIF-1 activation and downstream genes such as VEGF using macrophage cells, which is expected to inhibit the systemic inflammatory response to surgery (64). In terms of tumor-promoting effects, propofol has been found to increase the migration of breast cancer cells in association with the activation of the γ-aminobutyric acid type A receptor (65), and to promote the proliferation and migration of human breast cancer cells in association with the inhibition of p53 and activation of nuclear factor E2–related factor-2 *in vitro* (66). The discrepant effects of propofol on breast cancer may be due to the heterogeneity of this type of cancer; propofol may act differently on different types of cancer cell. In addition, the findings may reflect the lack of standardization of experimental parameters such as the propofol concentration and duration of exposure to cancer cells.

### Local Anesthesia

Clinically relevant concentrations of lidocaine, the most commonly used local anesthetic, enhance NK cell activity *in vitro via* the release of lytic granules in a variety of human leukemia cells (57). Local anesthetics inhibit the growth of several types of cancer cell, but the mechanism of action is unknown. These anesthetics block voltage-gated sodium channels (67), which are highly expressed in breast cancer and involved in the metastatic process (68). Local anesthetics that cause channel blockade inhibit tumor growth. Indeed, lidocaine inhibits tumor cell proliferation and differentiation *in vitro*, exhibits cytotoxicity against mesenchymal stem cells, and may inhibit tumor growth and metastasis (69). Clinically useful concentrations of lidocaine induced the apoptosis of breast cancer cells *in vitro* and *in vivo*, suggesting the usefulness of LA for breast cancer surgery (70). Lidocaine, which inhibits the kinesin motor protein, also decreases the formation and function of tubulin microtentacles *in vitro*, suggesting that it has a novel ability to inhibit breast cancer metastasis (71). The use of lidocaine at clinical concentrations *in vitro* causes DNA demethylation as a tumor-suppressive effect on ER-positive and -negative breast cancer cells (72). In addition, lidocaine was shown to inhibit the growth of luminal, TN, and human epidermal growth factor receptor 2 (HER-2)–positive breast cancer cell lines *in vitro*, the migration of breast tumor epithelial cells relative to normal breast epithelial cells, and the anchorage-independent growth of TN breast cancer cells (73). The intraperitoneal administration of lidocaine improved the survival of mice injected intraperitoneally with TN breast cancer cells at doses comparable to those used for analgesia in current clinical practice (73). These results suggest that clinically relevant concentrations of lidocaine directly inhibit the growth and metastasis of breast cancer cells. Other studies have shown that the systemic administration of amide local anesthetics inhibits the biological properties of cancer cells (74, 75); thus, the systemic administration of lidocaine at the time of tumor resection may inhibit cancer progression. In a mouse model of breast cancer, combination lidocaine and sevoflurane (but not ketamine) anesthesia suppressed lung metastasis, possibly due to the anti-inflammatory and anti-angiogenic effects of lidocaine (74). Similarly, in a mouse model of 4T1 breast cancer with surgery performed under sevoflurane anesthesia, the combined administration of cisplatin and lidocaine significantly reduced lung metastasis compared with the control and the administration of cisplatin alone, but did not reduce liver metastasis compared with the control (75). The serum VEGF and IL-6 levels did not differ significantly among these groups, suggesting that lidocaine enhances the metastasis-inhibiting effect of cisplatin under sevoflurane anesthesia (75).

The plasma concentrations of systemically administered lidocaine (as IVA) are significantly higher than those achieved with RA, but not LA. Furthermore, a recent study showed that perioperative lidocaine IVA reduces the postoperative extracellular trapping of neutrophils, an immune and angiogenic factor, and the postoperative expression of MMP-3 in patients undergoing breast cancer surgery, regardless of the GA technique (76). These results suggest that the intravenous administration of lidocaine at the time of breast cancer surgery reduces the risk of postoperative recurrence.

The local anesthetic ropivacaine has a breast cancer–inhibiting effect *in vitro* due to the disruption of mitochondrial function (77). It inhibited the phosphorylation of Akt, mechanistic target of rapamycin (mTOR), rS6, and ErbB3 binding protein 1 in breast cancer cells, suggesting a link between the Akt/mTOR signaling pathway and mitochondrial function in the context of breast cancer (77). This finding helps us to properly understand the mechanism by which local anesthetics reduce the risk of tumor recurrence. In another study, several local anesthetics (bupivacaine, levobupivacaine, and chloroprocaine) had different *in-vitro* effects on breast cancer cell survival and migration, suggesting that these effects depend on the exposure time, anesthetic type, and cell line (78).

### Muscle Relaxants

Muscle relaxants are often used for GA. Increases in doses of the chemical reference substances rocuronium bromide and suxamethonium chloride decreased the numbers of normal breast epithelial cells and hormone receptor (HR)-positive breast cancer cells, but not TN breast cancer cells, *in vitro* (79). Furthermore, rocuronium bromide promoted the invasion, adhesion, and proliferation of TN breast cancer cells, whereas vecuronium bromide had no significant effect on breast cancer cell motility or invasion (79). These findings suggest that certain muscle relaxants affect breast cancer progression.

### Mechanical Ventilation

The use of mechanical ventilation during cancer surgery has been hypothesized to promote lung metastasis; in a mouse model, it altered the interstitial and tissue environments of the lung to favor tumor formation (80). The mechanical ventilation of mice implanted with breast cancer cell lines during MT under GA significantly increased the number of circulating breast cancer cells remaining in the lung microvasculature and the occurrence of postoperative lung metastasis (80). Immunohistochemical analysis showed increased infiltration of CD68-positive macrophages in the injured lung parenchyma and metastatic tumors, and increased expression of epithelial cell adhesion molecules in metastatic nodules (80). Lung metastasis induced by mechanical ventilation occurs *via* the attraction of circulating tumor cells (CTCs) to the site of lung injury and promotion of the growth of existing lung micrometastases (80). In addition, the paracrine secretion of pro-inflammatory cytokines may induce metastasis to organs other than the lung (81). These observations suggest that the metastasis-promoting effects of mechanical ventilation during breast cancer surgery under GA need to be considered. Non-intubated metastasectomy with video-assisted thoracic surgery induces fewer inflammatory and immune reactions than does conventional surgery with intubation under GA (82). Moreover, with the de-escalation of breast cancer surgery, outpatient procedures can be performed without mechanical ventilation, with the use of lidocaine LA, low-dose propofol IVA, and/or midazolam sedation, which may reduce the recurrence rate; however, this evidence derives from retrospective cohort studies, not studies involving comparison with alternative anesthetic techniques such as standard GA (83, 84). In addition, awake surgery for breast cancer with LA causes less postoperative lymphopenia and may reduce the risk of tumor progression relative to GA (7). Further RCTs comparing total intravenous anesthesia (TIVA) or inhalation anesthesia with mechanical intubation with propofol IVA and/or sedation are needed to clarify the effect of mechanical ventilation on breast cancer recurrence after BCS.

## Possible Mechanisms by Which Surgical Stress and Anesthesia-Induced Immunosuppression Promote Distant Metastasis in Patients With Breast Cancer

As most breast cancer surgeries can consist of BCS with axillary management (e.g., SLNB or ALND), the impact of surgical stress on immunosuppression can be limited. MT with ALND may cause more surgical stress, leading to immunosuppression, and increase breast cancer recurrence relative to BCS with SLNB. Similarly, the use of inhalation anesthesia and opioids during breast cancer surgery can lead to immunosuppression, increasing recurrence and decreasing survival rates. Decreased host immunity may promote the growth of residual tumor cells in the surgically resected area, dormant tumor cells in other organs, and CTCs after surgery.

Breast cancer is a systemic disease; at the time of initial diagnosis, cells released from the primary tumor are circulating and present as micrometastases (85). In the perioperative period, breast cancer cells may escape surveillance by components of the innate and adaptive immune responses, such as NK cells and CTLs, which promotes distant metastasis *via* angiogenesis. Tumor dormancy, a quiescent state, is not well understood clinically; it is considered to comprise the lack of angiogenesis and tumor–host immunological equilibrium (86). Under perioperative immunosuppression, dormant cancer (stem) cells may reawaken and regenerate, and they may be detected as clinically visible foci months or years after surgical resection despite adjuvant treatment (86).

Cancer cells produce an immunosuppressive network of tumor-derived soluble factors (TDSFs), such as VEGF, which in turn recruit myeloid-derived suppressor cells (MDSCs), which are involved in CMI suppression, from the bone marrow (87). In the tumor microenvironment, immunosuppression due to the use of inhalational anesthesia may suppress the anti-metastatic effects of CMI and allow cancer cells to spread, affecting cancer recurrence and long-term outcomes. Immunosuppression induced by TDSFs can affect residual tumor cells and existing micrometastases and may lead to the formation of new metastatic foci (6).

## Effect of Anesthetics on Tumor Angiogenesis, Immune Function, Inflammation, and the Clinical Outcomes of Breast Cancer Surgery

Sevoflurane is thought to promote angiogenesis, whereas propofol inhibits it. Compared with inhalation anesthesia with sevoflurane, TIVA with propofol/remifentanil effectively inhibited the release of VEGF-C induced by breast cancer surgery, but did not significantly affect the 2-year RFS rate, suggesting that it does not affect short-term breast cancer recurrence (88). Because propofol is less immunosuppressive than inhalation anesthetics, it induces changes in immune cells (e.g., Tregs, Th1 and Th17 cells, NK cells, and CTLs) during breast cancer surgery comparable to those induced by sevoflurane, suggesting that anesthetics have minimal effects on perioperative immune activity (89). The effect of propofol on breast cancer recurrence needs to be investigated further, such as in an RCT comparing the use of RA and TIVA with propofol for anesthesia in breast cancer surgery.

MDSCs are immunosuppressive myeloid cells, and the number of these cells present is related closely to the breast cancer stage, clinical treatment response, and prognosis. Anesthesia with sevoflurane and propofol did not significantly alter the number of MDSCs or the prognosis after breast cancer surgery; compared with BCS, MT with a high degree of surgical stress reduced the number of MDSCs but did not significantly alter the prognosis (90). The postoperative presence of CTCs may be an independent factor influencing long-term outcomes in patients with breast cancer. In an RCT, the type of anesthesia (sevoflurane or propofol) did not affect the number of CTCs present over time after breast cancer surgery, but sevoflurane use significantly increased the maximum number of tumor cells postoperatively (91). In addition, NK cell activity was not associated with the number of CTCs (91).

In another RCT, balanced GA with opioid analgesia increased MOR expression, but not the expression of the immune cell markers CD56, CD57, CD4, and CD68, in resected breast tumors relative to paravertebral-propofol anesthesia (92). Propofol use may be superior to the use of inhalation agents for anesthesia during breast cancer surgery in terms of host defense immunity, but it did not alter the immune response (in terms of NK cells, CTLs, TNF-α, IL-6, and IL-10) or the apoptosis rate relative to sevoflurane in co-culture with a breast cancer cell line (93).

Inflammation and immunosuppression due to the elevation of the neutrophil-to-lymphocyte ratio (NLR) reflect breast cancer progression and adverse outcomes. In one study, the postoperative (but not preoperative) NLR was lower in the paravertebral propofol group than in the inhalation anesthesia and opioid groups (94), suggesting that paravertebral-propofol anesthesia inhibits the postoperative NLR elevation that may lead to breast cancer recurrence. In addition, NSAIDs may reduce breast cancer recurrence and act on biological mechanisms present in overweight patients. A retrospective study showed that the intraoperative administration of ketorolac was associated with significantly less distant recurrence than was diclofenac administration in patients with high body mass indices undergoing breast cancer surgery (95).

In an RCT, pectoral nerve II block under GA during breast cancer surgery increased the percentage of peripheral NK cells, NK cell–killing activity, and plasma IL-2 level postoperatively relative to GA (96). These results suggest that pectoral nerve II block had a lesser immunosuppressive effect than GA, thereby improving immunity. In another study, propofol-remifentanil anesthesia and postoperative ketorolac analgesia increased NK cell cytotoxicity relative to baseline, whereas sevoflurane-remifentanil anesthesia and postoperative fentanyl analgesia decreased this cytotoxicity, adversely affecting immune function, in patients undergoing breast cancer surgery (97).

## Retrospective Studies

Thirteen retrospective studies on anesthetic techniques and breast cancer recurrence have been reported (**Table 1**). Inhalation GA has been compared with RA techniques such as PVB-based GA (98, 99, 101–103), intravenous propofol–based GA (100, 104, 105, 107–110), and LA and propofol-based anesthesia (106). In two of these studies, recurrence rates were lower and RFS rates were higher in patients who underwent MT with RA or IV propofol–based GA than in those who underwent the procedure with inhalation-based GA (98, 104). In addition, reduced recurrence and increased survival were observed with RA or intravenous propofol–based GA than with inhalation-based GA for BCS and MT in three studies (99, 100, 110). Propensity score matching with the same variables was used in seven studies, of which one showed a potential benefit of propofol anesthesia (110). These findings suggest that RA and intravenous propofol–based GA reduce breast cancer recurrence compared with inhalation GA. However, the sample size and follow-up period were insufficient to assess breast cancer recurrence in some of the studies.

**Table 1:** Retrospective analyses of anesthetic technique and breast cancer recurrence.

Ref. (year)	Cancer type (patient *n*)	Surgery type	Anesthetic technique	Outcomes	Benefit/remarks
98 (2006)	Stage I–III breast (*n* = 129)	Mastectomy and axillary clearance	GA/PVA (*n* = 50) *vs.* GA/opioid anesthesia (*n* = 79)	4-fold reduced recurrence or metastasis risk during 2.5 to 4-year follow-up period with GA/PVA Increased RFS at 3 years with GA/PVA (94% vs. 77%)	Positive
99 (2014)	Stage 0–III breast (*n* = 619)	Breast-conserving surgery or total mastectomy	RA (*n* = 123) *vs.* RA/GA (*n* = 90) *vs.* GA (*n* = 406)	Trend of reduced recurrence with RA, with or without GA	Potential benefit
100 (2014)	Breast, colon, rectal (*n* = 2838)	Radical cancer surgery	Propofol (*n* = 902) *vs.* sevoflurane (*n* = 1935)	Favorable 1- and 5-year OS rates with propofol	Potential benefit
101 (2015)	Stage 0–III breast (*n* = 358)	Partial or total mastectomy without axillary node dissection	GA/PVA (*n* = 193) *vs.* GA (*n* = 165)	No difference in recurrence	Negative
102 (2016)	Stage 0–III breast (*n* = 1107)	Mastectomy or breast-conserving surgery	LRA (*n* = 646) *vs.* GA (*n* = 461); PSM (*n* = 375 each)	No difference in OS, DFS, or LRR	Negative/PSM
103 (2016)	Stage I–III breast (*n* = 792)	Mastectomy with or without axillary node dissection	PVB (*n* = 198) *vs.* opioid-based analgesia (*n* = 594); PSM (*n* = 197 each)	No difference in RFS or OS	Negative/PSM
104 (2016)	Stage I–III breast (*n* = 325)	Modified radical mastectomy	Propofol TIVA (*n* = 173) *vs.* sevoflurane (*n* = 152)	Less recurrence over 5 years with propofol	Positive
105 (2017)	Stage I–III breast (*n* = 2645)	Breast-conserving surgery or mastectomy	Propofol TIVA (*n* = 56) *vs.* inhalation anesthesia (*n* = 2589): PSM (1:5 matching for each inhalation agent)	No difference in RFS or OS	Negative/PSM
106 (2017)	Stage I–II breast (*n* = 91, elderly)	Breast-conserving surgery with SLNB or axillary dissection	LA/midazolam/remifentanil/propofol (*n* = 37) *vs.* GA (*n* = 54)	No difference in locoregional RFS or OS	Negative
107 (2019)	Stage 0–III breast (*n* = 976)	Breast cancer surgery	Propofol (*n* = 344) *vs.* desflurane (*n* = 632); PSM (*n* = 296, 592)	No difference in LRR or 5-year OS	Negative/PSM
108 (2019)	Stage 0–III breast (*n* = 5331)	Breast-conserving surgery or total mastectomy	Propofol TIVA (*n* = 3085) *vs.* inhalation anesthesia (*n* = 2246); PSM (*n* = 1766 each)	No difference in 5-year RFS or OS	Negative/PSM
109 (2020)	Stage 0–III breast (*n* = 1026)	Mastectomy	Propofol TIVA (*n* = 814) *vs.* sevoflurane (*n* = 212); PSM (*n* = 159 each)	No difference in 1-year RFS HR for recurrence or metastasis after sevoflurane vs. propofol was significantly higher for luminal B HER-2(+) subtype than for other subtypes	Negative/PSM
110 (2020)	Stage 0–IV breast (*n* = 6305)	Total or partial mastectomy, with or without axillary clearance	Propofol (*n* = 3296) *vs.* sevoflurane (*n* = 3209)	Trend toward better 5-year OS with propofol	Potential benefit/PSM

GA, general anesthesia; PVA, paravertebral anesthesia; RFS, recurrence-free survival; RA, regional anesthesia; OS, overall survival; LRA, local or regional anesthesia; PSM, propensity score-matched analysis; DFS, disease-free survival; LRR, locoregional recurrence; PVB, paravertebral block; TIVA, total intravenous anesthesia; SLNB, sentinel lymph-node biopsy; LA, local anesthesia; HR, hazard ratio; HER-2, human epidermal growth factor receptor 2.

Two meta-analyses including data on breast cancer and other cancers have been reported (**Table 2**). One meta-analysis showed no OS or RFS benefit of GA/RA over inhalation GA for gastrointestinal, prostate, breast, and ovarian cancer surgeries (111). The other meta-analysis showed that propofol-based TIVA for breast, esophageal, and non-small cell lung cancer surgeries (thus not breast cancer surgery alone) was associated with improved OS and RFS relative to inhalation anesthesia (112). Thus, intravenous propofol–based GA, but not GA/RA, may reduce breast cancer recurrence and increase survival compared with inhalation GA.

**Table 2:** Meta-analyses of anesthetic technique and breast cancer recurrence.

Ref. (year)	Cancer type (patient *n*)	Surgery type	Anesthetic technique	Outcomes	Benefit/remarks
111 (2017)	Gastrointestinal, breast, prostate, ovarian (*n* = 67,577)	Cancer surgery	RA/inhalation anesthesia vs. inhalation anesthesia	No difference in OS, RFS, or BRFS Some benefit of OS in RCT on colorectal cancer	Negative
112 (2019)	Breast, esophageal, NSLC (*n* = 7866) Breast, colorectal, gastric, esophageal, NSLC, mixed (*n* = 18,778)	Radical cancer surgery	Propofol TIVA vs. inhalation anesthesia	Improved RFS with TIVA Improved OS with TIVA	Positive

RCT, randomized controlled trial; NSLC, non-small cell lung cancer; BRFS, biochemical recurrence-free survival.

## Prospective Studies

Two prospective RCTs examining anesthetic techniques and breast cancer recurrence have been reported (**Table 3**). In the first trial, the use of standardized GA alone, GA plus single-injection thoracic paravertebral block (TPVB), and GA plus TPVB for 72 continuous hours was compared in a total of 180 patients with breast cancer undergoing modified radical MT (113). Neither TPVB technique had a major effect on postoperative local recurrence, metastasis, or 5-year mortality (113). The sample size and follow-up period in that study were insufficient for comprehensive evaluation of the effect of PVB on breast cancer recurrence. In the second RCT, breast cancer recurrence at a median of 36 months did not differ according to the use of PVB/propofol-based GA or sevoflurane/opioid-based GA in a total of 2108 patients who underwent surgery for breast cancer (114). That study was designed based on a retrospective report that GA/PVB reduced breast cancer recurrence at 3 years postoperatively by about one-fourth compared with GA/opioid use in patients who underwent MT with ALND (98), but it did not yield the same results. Several factors may explain this discrepancy. First, there was a large overlap in the use of propofol, sevoflurane, and opioids in both groups in the prospective study. Second, patients in that study did not undergo MT, and more than 30% of BCSs included were performed in China. Third, the median follow-up period was insufficient, as >50% of HR-positive breast cancers recur at >5 years postoperatively. Fourth, the frequency of breast cancer recurrence depends on the tumor subtype; TN and HER-2-positive breast cancers are more likely to recur than are HR-positive breast cancers. However, the randomization variables used in the prospective study pertain only to ER status. These factors may have led to bias and prevent the drawing of an accurate conclusion regarding the effect of PVB on breast cancer recurrence.

**Table 3:** Prospective randomized trials on anesthetic technique and breast cancer recurrence.

Ref. (year)	Cancer type (patient *n*)	Surgery type	Anesthetic technique	Outcomes	Benefit/remarks
113 (2017)	Stage I–IV breast (*n* = 180)	Modified radical mastectomy	GA (*n* = 58) vs. GA with single-injection TPVB (*n* = 56) vs. GA with continuous TPVB for 72 h postoperatively (*n* = 59)	Little to no effect of TPVB on local recurrence, metastasis, or mortality at 5 years	Negative
114 (2019)	Stage 0–III breast (*n* = 2132)	Breast cancer surgery	RA/propofol (*n* = 1043) vs. sevoflurane/opioids (*n* = 1065)	No difference in recurrence at a median of 36 months	Negative

TPVB, thoracic paravertebral block.

Three prospective RCTs (one completed and two ongoing) have been designed to investigate the relationship between anesthesia technique and breast cancer recurrence. A pilot trial (NCT01975064) examined the effects of propofol IVA and sevoflurane anesthesia on survival after radical surgery in patients with breast, colorectal, prostate, melanoma, lung, and other cancers; of 217 eligible patients, 146 were recruited (67.3% recruitment rate), supporting the performance of a large RCT to determine the effect of anesthetic technique on cancer recurrence (115). In the second trial (NCT04074460), the efficacy of propofol IVA and inhalation anesthetics such as sevoflurane, isoflurane, and desflurane is being compared in terms of recruitment (75%) and anesthesia administration (90%) success rates among eligible patients with breast, colorectal, prostate, lung, melanoma, and other cancers. In the third trial (NCT01916317), the effects of the perioperative injection of lidocaine in the setting of breast cancer are being examined as part of the assessment of the *in-vivo* ability of local anesthetics to reduce the dissemination of cancer cells during surgery and improve the disease-free interval (i.e., affect tumor recurrence). Future RCTs must be designed with consideration of the breast cancer surgery type and use of mechanical ventilation, as the use of less-immunosuppressive anesthesia and non-mechanical ventilation may best reduce breast cancer recurrence. For patients who have undergone MT with SLNB or ALND, the effects of propofol IVA with RA and inhalation anesthesia with opioids could be compared. For patients who have undergone BCS with SLNB, the effects of mechanical and non-mechanical ventilation could be compared.

## Concluding Remarks

At this time, RCTs have not provided sufficient evidence that the anesthetic technique is associated with the recurrence rate or long-term outcomes in patients undergoing breast cancer surgery. Preclinical and clinical studies have provided conflicting data on the effects of inhalation anesthetics, propofol, and opioids on the immune response and breast cancer growth. However, RA (e.g., PVB or propofol IVA), LA, and/or non-mechanical ventilation with non-opioid anesthesia may reduce breast cancer recurrence compared with intravenous or inhalation GA, opioid use, and/or mechanical ventilation. As most current breast cancer surgeries, especially BCS, are performed with IVA, the superiority of this technique to inhalation anesthesia may be difficult to evaluate. Nevertheless, such efforts are being made in ongoing RCTs, and we await their results for breast cancer and other cancers. Further such trials are needed for the development of systemic breast cancer therapies, which will bring us closer to a cure for primary breast cancer.

## Author Contributions

Conceptualization, RK. Resources, RK. Writing—original draft preparation, RK. Writing—review and editing, RK, AK, MW, and TK. All authors contributed to the article and approved it for publication.

## Conflict of Interest

The authors declare that the research was conducted in the absence of any commercial or financial relationships that could be construed as a potential conflict of interest.

## Publisher’s Note

All claims expressed in this article are solely those of the authors and do not necessarily represent those of their affiliated organizations, or those of the publisher, the editors and the reviewers. Any product that may be evaluated in this article, or claim that may be made by its manufacturer, is not guaranteed or endorsed by the publisher.

## Abbreviations

ALND, axillary lymph node dissection; ARF6, adenosine diphosphate–ribosylation factor 6; BCS, breast-conserving surgery; CMI, cell-mediated immunity; CTC, circulating tumor cell; CTL, cytotoxic T lymphocyte; DEX, dexmedetomidine; ER, estrogen receptor; ERK, extracellular signal–regulated kinase; GA, general anesthesia; HER-2, human epidermal growth factor receptor 2; HIF-1α, hypoxia-inducible factor-1α; HPA, hypothalamic–pituitary–adrenal; HR, hormone receptor; IL, interleukin; IVA, intravenous anesthesia; LA, local anesthesia; MDSC, myeloid-derived suppressor cell; miR, micro RNA; MMP, matrix metalloproteinase; MOR, μ-opioid receptor; MT, mastectomy; mTOR, mechanistic target of rapamycin; NF-κB, nuclear factor κB; NK, natural killer; NLR, neutrophil-to-lymphocyte ratio; NSAID, nonsteroidal anti-inflammatory drug; OS, overall survival; PGE_2,_ prostaglandin E_2_; PVB, paravertebral block; RA, regional anesthesia; RCT, randomized controlled trial; RFS, recurrence-free survival; SLNB, sentinel lymph node biopsy; SNS, sympathetic nervous system; TDSF, tumor-derived soluble factor; Th, T helper; TIVA, total intravenous anesthesia; TN, triple negative; TNF-α, tumor necrosis factor-α; TPVB, total paravertebral block; Treg, regulatory T cell; VEGF, vascular endothelial growth factor.
